# Body dysmorphic disorder, dysmorphophobia or delusional disorder—somatic subtype?

**DOI:** 10.4103/0019-5545.31561

**Published:** 2006

**Authors:** V.K. Aravind, V.D. Krishnaram

**Affiliations:** * Consultant Psychiatrist, Ram Psychiatry Hospital and Institute, Madurai 625020, Tamil Nadu; ** Professor of Psychiatry (VRS), Chief Psychiatrist and Director, Ram Psychiatric Hospital Institute, Madurai 625020, Tamil Nadu

**Keywords:** Body dysmorphic disorder, change of face appearance, delusional disorder, somatic type

## Abstract

Excessive concern about the appearance of one's body is the hallmark of body dysmorphic disorder (BDD). A case with recurrent intrusive preoccupation and concern about the appearance of the face, ritualistic behaviours associated with this preoccupation, resulting in social and interpersonal difficulties is presented. The difficulty to draw a discrete boundary between BDD and a delusional disorder of somatic type is highlighted.

## INTRODUCTION

Body dysmorphic disorder (BDD) previously known as ‘dysmorphophobia’ is defined as a preoccupation with an imagined defect in one's physical appearance. The preoccupation is associated with many time-consuming rituals such as mirror gazing or constant comparing.^1^ One of Freud's patients who was subsequently analysed by Brunswick was known as the ‘Wolfman’ and he was preoccupied with imagined defects on his nose.^2^

In 1886, Morselli described dysmorphophobia. Dysmorphophobia by proxy was reported by R. Laugharne in 1997—the patient was preoccupied not with her own appearance but how her potential offspring might look.^3^

There is frequent comorbidity in BDD, especially in depression, social phobia, and obsessive–compulsive disorder (OCD) and delusional disorder.^4^ Beliefs about defects in appearance usually carry strong personal meanings and implications. A belief that his nose was too big caused one patient to feel that he would end up alone, unloved and that he might look like a crook. Also, such patients are likely to display delusions of reference, believing that people around them notice their defect and evaluate them negatively or humiliate them as a consequence of their ugliness.^5^

A further aspect of BDD is time-consuming behaviours adopted by sufferers to examine the defect repeatedly or to disguise or improve it. Examples include gazing into the mirror to compare particular features with those of others; and some other features such as excessive grooming, which can be quite deleterious especially where the skin is concerned, camouflaging the defect with clothes or make-up, dieting and pursing dermatological treatment or cosmetic surgery.

Delusional disorder comprises a heterogeneous group of disorders of unknown aetiology whose hallmark and chief features are the presence of a single delusional system. Major modes of presentation of somatic delusional disorder, ‘mono-symptomatic hypochondrical psychosis’ are those of infestations by insects, worms and foreign bodies, emitting a foul odour (halitosis) or of being ugly.^6^

## THE CASE

A 37-year-old well-educated male from the middle socio-economic class was presented with a belief that his face was changing into one of an ‘eunuch’ with a perceived emphasis on his face, lips, ears and his voice in articulation and also people commenting him as eunuch (number nine)—nine is a social–cultural term given to an eunuch. The total duration of his illness was 7 years. The illness probably started in 1997, while he was at his workplace, talking with his customers. He suddenly started feeling that his customers were referring and talking about him as an ‘eunuch’ by stressing on the words ‘nine’ boldly and repeatedly. As days passed, he was excessively preoccupied with his face changing into ‘eunuch’-like appearance. Change of face into that of ‘eunuch’ was noticed by other people and his own family members—wife, mother and others—with an impact of mostly avoiding him. He felt that they noticed his ‘eunuch’ face with disgust and shame. He could not continue his job and resigned it as he was observed by each and every person around him pointing out purposefully to him with words nine and similar numbers. He felt shameful to leave his home, to mingle with others and to have social interaction. He felt uneasy and was preoccupied with his imagined eunuch facial appearance.

During the course of his illness he became more pre-occupied, suspicious and started spending a great deal of time in front of the mirror. Where he once used to spend 10 minutes for his shave, after his illness he would spend half an hour to one hour before the mirror gazing for the imagined change in his face. Similarly, while combing his hair he would spend excessive time. Because of this preoccupation, he had significant distress, impairment in his social, occupational and other important areas of functioning.

All these years he had a feeling of fear and sadness along with above symptoms. Initially, he experienced suicidal ideas but never attempted. He had sleep disturbances too for the past 6 years, but with medications he maintained his sleep fairly well. During these 7 years he had consultations from different psychiatrists and an andrologist. He was put on imipramine, clomipramine, fluoxetine, dotheipin at different points of his illness. To some extent his symptoms were relieved.

A detailed history revealed that he could note the foul odour from his own body (?halitosis), and that his friends too could smell it, and that it was the odour of semen. This symptom lasted for 6–8 months. He had a consultation from a physician for this complaint and the physician reassured him that there was no foul odour from his body. The reason for the above symptom was interpreted by the physician as a result of his habit of excessive masturbation.

Psychiatric examination revealed delusions of reference, somatic delusions (monosymptomatic hypochondrical delusions), impaired judgement and insight. There were no bizarre delusions or hallucinations or other first rank symptoms of schizophrenia. EEG and CT brain were normal and non-contributory. Psychometric assessment with Rorschach inkblot test did not reveal any indicators of schizophrenic thinking or perception. The patient was started on olanzapine 5 mg for 2 weeks and subsequently the dose was increased to 10 mg and 15 mg, and was maintained at 15 mg for 4 weeks. After 2 months of starting the treatment, the patient had only minimal improvement.

## DISCUSSION

Patients with BDD have an excessive preoccupation with a slight or imagined defect of a specific body part and that it results in impaired social, academic or occupational functioning. In this case, the patient had an excessive preoccupation that his face was changing into one of an eunuch, which restricted him from going to work and he became housebound. He was convinced that he had a defect in his physique, particularly his face.

Persons who have BDD spend many hours focusing on their physical features and engaging themselves in repetitive and time-consuming behaviours. Classical descriptions of spending excessive time before the mirror during shaving, combing and checking in reflective surfaces and consistently seeking reassurance from the physicians form the hallmark of BDD. Studies have shown that patients of BDD typically think about their perceived deformity for at least an hour (the mean time is 3–8 hours a day).^7^ This is true in this particular case with an average spending time of 1 hour standing in front of the mirror and getting himself reassured of his imagined eunuch appearance of the face.

Individuals with BDD can receive an additional diagnosis of delusional disorder—somatic type, if their preoccupation with an imagined defect in appearance is held with a delusional intensity.^8^ Approximately 50% of patients with BDD meet the criteria in DSM-IV for a delusional disorder, somatic type. De Leon *et al.* point out the difficulties in determining whether a dysmorphophobia is delusional or not.^9^ Phillips and Hollander state that in BDD, it appears that a continuum exists between preoccupation and delusion and thus it is difficult, if not impossible, to draw a discrete boundary between BDD and delusional disorder, somatic type. Furthermore, individual patients seem to move along the continuum.^10^

Debate continues as to whether the BDD is a discrete disorder. It is variously argued that it represents a variant of major disorders including social phobia, mood disorder, OCD, hypochondriasis and psychosis, particularly delusional disorder. These aspects are well illustrated in the present case.

Though the focus of patients with BDD is by definition on physical appearance, data exist on patients with BDD having obessional concerns about odour. Given the significant overlap between halitosis and BDD, one can postulate that halitosis is a variant of BDD and the diagnostic criteria of BDD should be extended to include odour.^11^ Whether halitosis or olfactory reference syndrome is truly a unique disorder, or merely a part of symptomatology of other psychiatric conditions remain controversial.^12^

Neuroleptic and antidepressant medications are re-commended as pharmcotherapy. Response to neuroleptic treatment has been suggested as a diagnostic test to distin-guish BDD from delusional disorder, somatic type.^13^ Delusional syndrome, in general, may respond to neuroleptics, whereas in BDD, even when the bodily preoccupation is psychotic, there is less likelihood of success.

## CONCLUSION

Delusional BDD can be double-coded as a delusional disorder and a BDD, a compromise that underscores the uncertainty about whether they are same or different disorders. An insight into BDD shows a spectrum from good to absent and may change over time, sometimes fluctuating between non-delusional and delusional thinking.
